# Editorial: User-centered approaches for designing assistive technologies to support older adults

**DOI:** 10.3389/fragi.2026.1864923

**Published:** 2026-07-28

**Authors:** Samuel Olatunji, Neziha Akalin, Xiaomei Liu, Renato Ferreira Leitão Azevedo

**Affiliations:** 1 Department of Industrial and Systems Engineering, Tickle College of Engineering, The University of Tennessee, Knoxville, TN, United States; 2 Department of Computer Science and Informatics, Jönköping University, Jönköping, Sweden; 3 Center on Aging Psychology, State Key Laboratory of Cognitive Science and Mental Health, Institute of Psychology, Chinese Academy of Sciences, Beijing, China; 4 Institute of Gerontology and Department of Epidemiology and Biostatistics, College of Public Health, University of Georgia, Athens, GA, United States

**Keywords:** assistive technologies, digital inclusion, healthy aging, older adults, service ecosystems, technology acceptance, user-centered

## 1 Introduction

For the first time in human history, global demographic patterns are shifting so that older adults will represent an increasingly larger share of the population. According to the United Nations World Population Prospects, 2024, by the mid-2030s, the number of people 80 and older is expected to reach roughly 265 million, exceeding the number of infants United Nations (2024). By the late 2070s, the population aged 65 and older is projected to reach approximately 2.2 billion, exceeding the number of children and adolescents under age 18. Many regions, including those with rapidly growing populations, are expected to experience substantial increases in both the proportion and the absolute number of older adults over the coming decades.

Digital and assistive technologies are an increasingly vital part of the response to these demographic pressures, offering opportunities to extend independence, support daily functioning, and relieve strain on formal care systems. Yet technology is not a standalone solution: its potential depends on how well it aligns with older users’ needs, capabilities, preferences, and living contexts. A well-designed system can enhance independence, safety, and quality of life for older adults Fisk et al. (2020). Designing effective assistive technologies, therefore, requires several considerations, including autonomy, confidence, ease of use, trust, acceptance, and sustained motivation, among other important factors. Key research challenges include designing supportive instructional systems, user-centered decision support and explainable intelligent systems, and intuitive, accessible interaction models that minimize cognitive load Zajicek (2005). Equally important are factors that influence longer-term engagement, which include perceived usefulness, social acceptability, privacy and security concerns, and the availability of ongoing technical and social support. Gaps in these areas continue to limit both initial adoption and sustained use of assistive technologies among older adults.

Furthermore, technology does not operate in a social vacuum MacLachlan and Scherer (2018). Assistive technologies are embedded within broader service systems, and well-designed solutions should account for the surrounding social and environmental infrastructure and factors. These factors should include care partners, living environments, healthcare providers, care settings and their workflow integration, technology developers and providers, as well as cultural contexts. The progress of assistive technology has been hampered by disconnected initiatives and activities, and this needs to be corrected MacLachlan et al. (2018). We should incorporate systems thinking as a way of thinking about the connections between things and how these are influenced by contextual and other factors. Part of the editorial’s goal is to encourage researchers, designers, providers, and users of assistive technology to think more systemically, so that we can advance more cohesive and resilient systems. User-centered approaches and considerations are central to systems thinking for assistive technologies.

The ten papers in this Research Topic address these concerns from complementary angles, synthesizing existing knowledge and extending it in new directions. To foster dialogue across contributions, we organize the Research Topic around five interrelated themes that capture recurring priorities for research and practice: (1) psychological and social determinants of acceptance and adoption; (2) learning, instructional design, and digital inclusion; (3) robotics, engagement, and institutional mediation; (4) assistive technologies as part of service systems; and (5) evidence, metrics, and emerging frontiers. Together, these papers provide a user-centered research agenda that aims at translating technological possibilities into meaningful outcomes for older adults.

## 2 Articles accepted in this Research Topic

Between 2024 and 2026, this Research Topic invited and received submissions addressing *User-Centered Approaches for Designing Assistive Technologies to Support Older Adults*. The Research Topic achieved an overall acceptance rate of 52.63%, with 10 of 19 submissions accepted (8 rejected at the full-manuscript stage and 1 declined at the abstract pre-submission stage). This edition brings together 43 authors from institutions across 5 countries (China, Italy, Malaysia, Norway, and United States). The purpose of this editorial is to synthesize the contributions of these ten accepted papers in relation to our original editorial vision. Of the accepted articles, seven were published in Frontiers in Public Health: Aging and Public Health, and three were published in Frontiers in Aging: Interventions in Aging. We provide a thematic synthesis of these articles in the following subsections.

### 2.1 Psychological and social determinants of acceptance and adoption

A core theme across the Research Topic is understanding why and how older adults adopt and interact with technology, with an emphasis on attitudes, emotional responses, and psychosocial effects. Zhang et al. examined the relationship between technological and environmental affordances, self-determination, and socioemotional affordances of age-appropriate smart wearables and older adults’ wellbeing, showing that perceptions of a device’s directiveness, interactivity, and visibility are associated with enhanced autonomy and satisfaction. This was an online study with 233 older adults aged 60 and above, in China, who use age-appropriate smart wearable devices. The findings argue for designs that go beyond functionality to support psychological needs that underpin quality of life.

Similarly, Liu and Kassim extended the Unified Theory of Acceptance and Use of Technology 2 (UTAUT2) to explore older adults’ intentions to use smart care applications for older adults in China. This was also conducted as online survey with 375 older Chinese adults aged 60 and over. They find that traditional predictors such as performance expectancy and effort expectancy coexist with psychological factors like trust, perceived risk, and life satisfaction in shaping behavioral intention, spotlighting the affective and cognitive landscape underlying technology uptake. Older adults differ in how they prioritize technological features, and understanding these preferences is essential for user-centered design.

Yang et al. combined qualitative insights with Kano modeling, a theoretical model for product development, UX and user-centered design, in order to explore which smart home functional quality attributes resonate with older users. This was conducted as an in-person semi-structured interview with 15 older adults aged 60 and above also from China. Their findings reveal age-related differences in feature priorities (e.g., different emphases on safety, daily living conveniences, or emotional support), offering practical design guidance for tailoring environments that older adults value.

### 2.2 Learning, instructional design, and digital inclusion

User-centered design must also address how older adults learn and engage with new systems, particularly in contexts of varied digital literacy. Ford et al. evaluate socioemotional learning strategies and instructional formats for older “digital immigrants” learning a new smartphone application. Across two online experiments, they examined older adults learning a new software application: 234 participants aged 55 and older used an instructional video (Experiment 1), and 141 participants aged 55 and older used a written manual (Experiment 2). These instructional materials were created without additional support, or to foster standard learning strategies (i.e., repetition or sentence generation of a novel information), or to encourage a socioemotional strategy (i.e., focus on the emotion related to the novel information). Their results show that video formats paired with socioemotional strategies yield superior learning outcomes compared to text manuals, underscoring that instructional design, such as the design, development, and organization of learning experiences, is an integral facet of accessibility beyond its content.

Ming et al. reviewed digital technologies in the context of rural aging in China, identifying opportunities and challenges related to infrastructure gaps, literacy barriers, and service availability. This was conducted as a systematic review that focused on the current application of digital technologies in the care of older adults in rural areas. Their systematic review included 26 studies, covering themes related to daily living needs, medical care needs, spiritual comfort needs, and cultural and recreational needs. This work highlights that user-centered design must consider digital inclusion at structural scales, especially in under-resourced and rural settings.

### 2.3 Robotics, engagement, and institutional mediation

Assistive technologies intersect with physical engagement and social connection, particularly in institutional settings. Preston et al. examined how robots can facilitate physical, cognitive, and social engagement in skilled nursing facilities (SNFs). The authors conducted an iterative design process involving 54 older adults and 5 care providers (three physical therapists, a director of rehabilitation, and an exercise scientist) in the United States to test robot-based activities, identify needs and challenges, and design prototypes for robot-supported interventions for SNF residents. They presented a variety of activities using seven different robots, including Paro, Stretch, Misty, Cozmo, PokerBot, NAO, and Quori. Their work emphasizes that robot design and deployment must account for social context, workflow integration, and the relational dynamics of care, highlighting how institutional settings shape the technology experience. A positive aspect of this publication is not only the range of robots used, but also the variety of activities presented. Different assistive technologies can support different task categories, including a range of activities of daily living, instrumental activities of daily living, and enhanced activities of daily living (see Olatunji et al.)

### 2.4 Assistive technologies as part of service systems

Another cluster of papers situates technology within broader service ecosystems and emotional experiences of care, moving beyond individual devices to how technologies operate within a network of services. Kattouw et al. used service design methods to co-create proposals for ecosystems that support older adults living at home. The study was conducted in Norway with multiple stakeholder groups (n = 58), including older adults aged 67 and above, from both urban and rural areas. Their work highlights that technologies must align with transportation, communication, and caregiving services to be meaningful elements in older adults’ everyday lives, underscoring that user-centered design also extends to service architecture itself.

Wu et al. contributed an emotional experience evaluation framework for Chinese smart care services, analyzing how older users in community settings emotionally engage with technology (such as smart devices, online software, and offline services) and care touchpoints. The study was conducted as a semi-structured interview including an emotional experience questionnaire survey with 28 older users, aged 62–85 years old, from three communities in Xi’an, China. Their model integrates affective responses into service improvement strategies, illustrating how user emotion can inform service system design.

### 2.5 Evidence, metrics, and emerging frontiers

Finally, rigorous evaluation and measurement frameworks are critical to understand the concrete impact of assistive technologies on older adults’ health and outcomes. In this regard, systematic reviews, meta-analyses, and bibliometric studies offer a structured, qualitative, and quantitative approach to synthesizing, organizing, and evaluating the existing literature within a research domain. Rather than serving as an end in themselves, these efforts aim to provide a comprehensive understanding of the current state of knowledge and to inform directions for future research and the development of practice guidelines and policies. Leo et al. provided a systematic review of Active and Assisted Living (AAL) technologies’ effects on psychosocial wellbeing, showing mixed evidence for outcomes such as quality of life, loneliness, depression, and fear of falling. Their review underscores methodological variability across studies and the need for shared evaluation standards. In a bibliometric mapping study, Zhong et al. illuminate global trends in gait analysis research related to mild cognitive impairment, identifying hotspots like dual-task walking and digital biomarkers. Such work situates assistive technologies within an evolving evidence base of sensors and analytics that may enable early detection and intervention.

## 3 Future research directions

Older adults are often described as skeptical toward assistive technologies, which may stem from factors such as cognitive decline, existing biases, or difficulties in learning new systems. However, when designed appropriately, technological solutions can support older adults in maintaining independence, accessing essential services, managing health outcomes, staying socially connected, and enhancing overall quality of life Fisk et al. (2020). To ensure these technologies meet users’ needs, user-centered design methods are essential. Despite growing interest, the current state-of-the-art in assisted living technologies for older adults still faces significant challenges in usability, acceptance, and long-term engagement.

This Research Topic focused on research that takes a user-centered approach for designing assistive technologies. The accepted papers used various approaches including online surveys Liu and Kassim; Zhang et al., systematic review Leo et al.; Zhong et al.; Ming et al., design workshops Kattouw et al., combination of multiple approaches (e.g., observations, questionnaires, semi-structured interviews) Wu et al.; Yang et al., iterative design process Preston et al., and empirical studies Ford et al. (2024).

Understanding aging and the needs of older adults with respect to supporting their health, wellness and quality of life is vital to reliably design assistive technologies that support the older adults. This includes identifying the potential of assistive technologies as well as their limitations in meeting these needs. User-centered design approaches that address one or more of the aforementioned challenges while leveraging the capabilities of the technologies to support health and wellness of the older adult are sought. In general, without proper design, the older adults may find it difficult to calibrate their trust in the system, thus influencing their willingness to adopt the continuous usage Zajicek (2005). As these systems become more intelligent and are capable of complex decision-making, it becomes increasingly important for the older adults to understand these systems better through user-focused design techniques that foster usability of the technology. The goal would therefore be to research into methods and design techniques that prioritize the older adults in the design of new technological capabilities, task performance processes, data storage and security options, privacy protection options, personalization of such systems to make them more usable and useful for the older adults without overwhelming them.

The assistive technologies investigated in this Research Topic included a medical smartphone application Ford et al. (2024), smart elderly care applications Liu and Kassim, assistive living technologies (i.e., home automation applications) Leo et al., service ecosystem for older adults living at home (including home care provision and age-friendly mobility) Kattouw et al., smart wearable devices Zhang et al., smart care service system Wu et al., smart home for older adults Yang et al., methods for gaits analysis in older adults with mild cognitive impairment Zhong et al., digital technologies for smart aging (i.e., mobile applications, websites and platforms, mobile devices and terminals, and virtual reality technologies, etc.) Ming et al., and robots Preston et al. We anticipated a greater number of submissions focusing on robotics, as there is a growing body of research focusing on robot usage in geriatric care settings Asgharian et al. (2022), particularly the interplay between physical embodiment, functionality, utility, participation, and social connection. Society is increasingly adopting intelligent agents—both embodied (e.g., robots) and disembodied (e.g., conversational agents and AI systems) across private, social, and institutional settings. As a result, it is essential to deepen our understanding of the complexities and nuances of interactions with these agents. Together, these contributions reflect the breadth of current research on user-centered approaches to designing assistive technologies, highlighting both practical implementations and methodological innovations aimed at improving the lives of older adults.

We also anticipated a large number of submissions related to health communication and health data visualizations, which unfortunately did not translate into any accepted publications. In our vision, that remains an important gap to be addressed Morrow et al. (2019); Nie et al. (2024). As digital health technologies increasingly serve as the interface mediating the communication of health information, such as clinical test results, appointments, prescriptions, mHealth applications (apps), and telehealth, it is important to consider user-centered gerontechnology design, addressing aspects related to both the medium (apps, websites, telehealth platforms) and the media, such as the content forms or communication materials (e.g., videos, health visualizations, text messages, reminders) through which these health communication messages are delivered.

**FIGURE 1:**
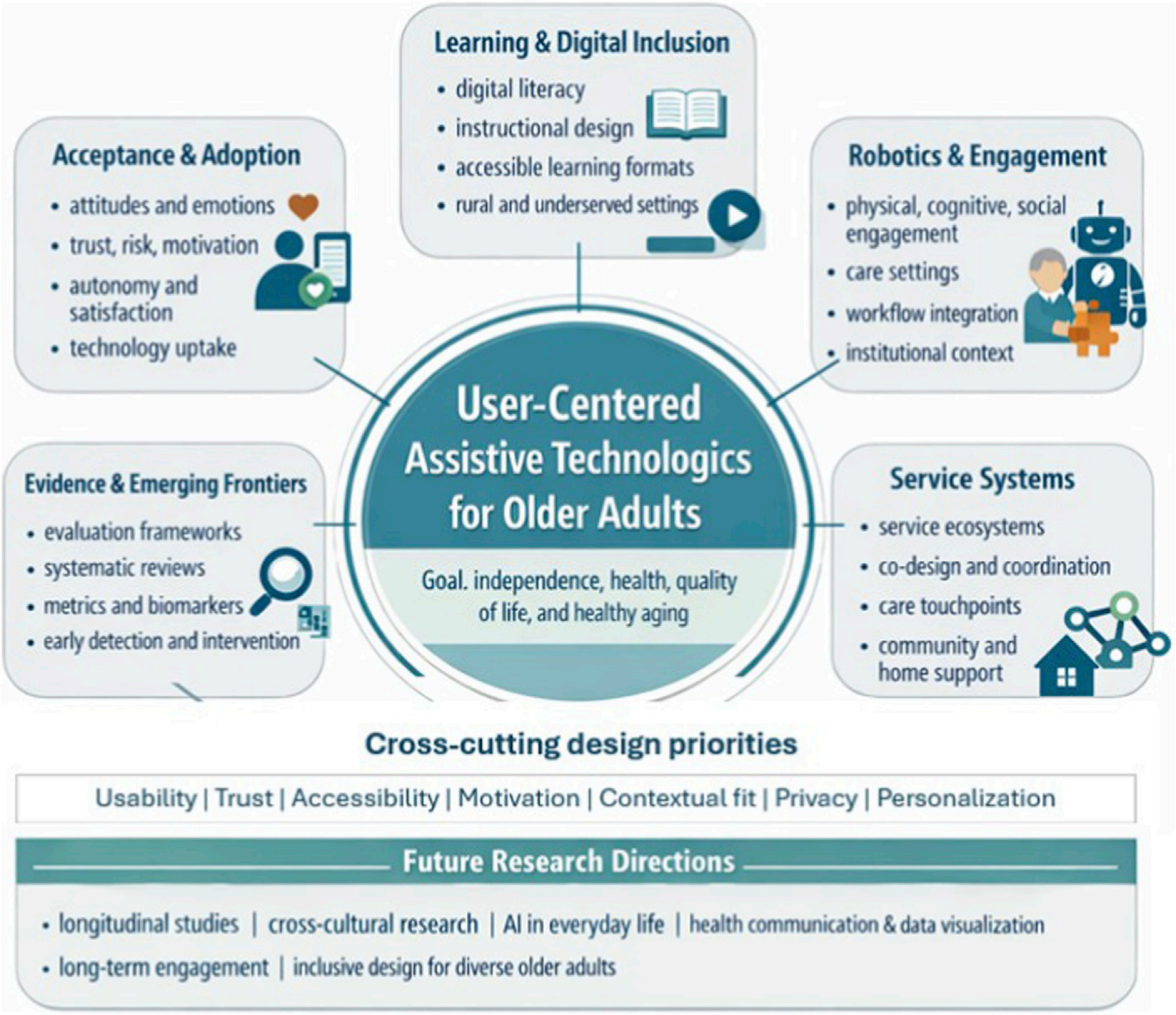
Summary of overall themes and future research directions. Image co-created with The University of Tennessee, AIHub, GPT-5.4.

The publications within this Research Topic collectively highlight several important directions for future research in assistive technologies for older adults (see Figure 1 for highlights of themes and future research directions).

Although the need for longitudinal studies has been widely acknowledged, such research remains scarce, limiting our understanding of long-term adoption, sustained engagement, and evolving perceptions of technology. Moreover, many studies are conducted within a single country or region, raising concerns about generalizability across cultural contexts; older adults from different cultural backgrounds may hold substantially different attitudes toward technology Akalin et al. (2021). Future research should therefore include more cross-cultural investigations and pay closer attention to variations in digital literacy, ensuring that technologies are designed for individuals with diverse levels of prior experience and confidence.

In addition, there is a clear gap regarding the role of AI in older adults’ everyday lives Lei and Chen (2025), in particular, how AI-driven systems influence autonomy, trust, decision-making, and how they may either alleviate or exacerbate digital cognitive barriers. Addressing these Research Topic requires longitudinal, mixed-method, and randomized designs that evaluate not only functional outcomes but also different social and emotional aspects including but not limited to: trust, perceived safety, emotional engagement, digital literacy, and social connectedness. Greater attention must also be given to the heterogeneity of older adults. It is important to recruit participants with different backgrounds such as different cognitive status, living contexts (home, rural, or institutional care), and digital experiences to support the development of adaptive, user-centered systems that align with socioemotional strengths and real-world needs.

Lastly, in many cases, the technology itself is not the primary barrier, rather, challenges arise from how the content delivered through these technologies is designed. Research Topic such as inaccessible or overly complex health data visualizations, unclear or poorly structured instructions, and information that appears unreliable or difficult to interpret can significantly limit the usability and effectiveness of otherwise functional systems. When the presentation and communication of information are not carefully designed with users in mind—particularly older adults—technologies intended to support health and wellbeing may instead create confusion, mistrust, or disengagement. This highlights the importance of focusing not only on technological capabilities but also on the clarity, accessibility, and reliability of the content conveyed through these systems. These directions are intended to provide a research roadmap for designing assistive technologies that effectively meet the needs of older adults.
